# In search of well-established evaluative criteria for the emerging qualitative methods of L2 affective variables

**DOI:** 10.3389/fpsyg.2022.995761

**Published:** 2022-08-25

**Authors:** Jiayi Du

**Affiliations:** College of Foreign Languages, Henan Institute of Science and Technology, Xinxiang, China

**Keywords:** affective variables, evaluative criteria, qualitative research, systematic qualitative, complex dynamic systems theory (CDST)

## Abstract

Qualitative research is marked by context sensitivity with an inclusion of the participants’ points of view and setting, how they can affect how participants feel and how this feeling is captured and interpreted by researchers (Yardley, 2017). This is the value of exploring affective variables through qualitative research. The present study focuses on the qualitative studies of L2 affective variables in recent years led by the complexity dynamic systems theory (CDST). This new line of research has employed innovative research methods compatible with the CDST, and has had useful findings. Yet, they seem to lack rigor and systematicity of research. Thus, this observed lack of consistency is problematized in the present study and attempts are made to set evaluative criteria for the judgment of the burgeoning studies and guiding the future line of qualitative and dynamic inquiry in the L2 affective domain. To this aim, the different sets of evaluative criteria proposed for qualitative research are derived from the mostly cited scholars in the research methodology. The relevance of each to the dynamic qualitative investigations of L2 affective variables is discussed. Then these evaluative criteria are abstracted and put forth for the qualitative research in the dynamic phase of second language acquisition (SLA) research. The overall goal is to guide future researchers with an interest in investigating the dynamic and developmental nature of L2 affective variables qualitatively. These evaluative criteria can pave the way for the emergence of more rigorous and systematic qualitative studies of L2 affective factors in the future.

## Introduction

Qualitative research is defined as an approach to social inquiry that deals with how we interpret and understand our experiences in our surrounding world (Holloway, 1997). Several different methods are subsumed under this overall approach to research (e.g., observations, interviews, and ethnography), yet they seek to make sense of the social reality of individual subjects, groups, or cultures (Fossey et al., 2002). The qualitative approach to research is generally used to explore the research participants’ cognitive, affective, attitudinal, or behavioral constructs. Overall, there are two distinctive paradigms to qualitative research: the positivist and interpretivist/pragmatic paradigms, with the latter being the predominant. As argued by Holloway (1997), all qualitative methods share an interpretive perspective on social reality especially in recent years with the warm reception of the social-constructivist perspective.

Different sources have been published to suggest different ways of carrying out qualitative research (e.g., Jacob, 1987; Tesch, 1990; Wolcott, 1990; Denzin and Lincoln, 1994; Creswell, 1998). Yet, they have not been exclusive to the second language acquisition (SLA) domain. The innovative emerging methods in the L2 affective domain in this review paper refer to the ones which have drawn the attention of researchers in this domain *via* a CDST perspective. It should be noted that all qualitative research methods do not necessarily incorporate a dynamic research perspective in the L2 affective domain (Dörnyei and Ryan, 2015). Thus, inspired by the CDST, some emerging research methods have been recently used in the L2 affective domain which are consistent with the CDST framework. Some of these emerging methods in the L2 affective domain are retrodictive qualitative modeling, the idiodynamic method, self-organizing maps, Q-methodology (Kruk et al., 2022), process-tracing (Yazdanmehr et al., 2021), ecological momentary assessment, and experience sampling method. What derived the application of these methods in the L2 affective domain is rooted in the problem-driven approach of CDST to inquiry which appreciates the expansion of the methodological repertoires in a given academic domain.

With respect to complex dynamic systems theory (CDST), Hiver and Al-Hoorie (2019) perceived the value of qualitative methods in that they facilitate fine-tuned observations of context-specific processes of development, which is, in recent years, more to the interest of SLA researchers. CDST in the field of SLA indicates that the process or the path of language learning is non-linear, context-bound and feedback sensitive (Dörnyei, 2014). Regarding the use of this meta-theory in SLA, Larsen-Freeman (1997) contended that SLA topics of research can significantly benefit from being explicitly investigated in the light of the CDST. Similarly, Larsen-Freeman and Cameron (2008) insightful and SLA domain-specific perspective on CDST is also considered one of the most prominent volumes published in SLA (de Bot, 2015). As SLA is an extensive and comprehensive domain hosting many other disciplines such as psychology, linguistics, and sociology, it is more often than not marked by an openness to the effect of external factors (Chapelle, 2014).

A broad influential re-orientation of the CDST has penetrated into the social sciences more extensively, bringing evidence for the fact that the majority of research topics in our time are characterized as dynamic and complex. This complexity and dynamicity need to be investigated with a compatible change in attitude to research (Capra and Luisi, 2014). Similarly, SLA has now become complex, and the conclusion that complexity has come about to affect SLA researchers’ attitudes to research should be warmly received (Hiver and Al-Hoorie, 2019). Corresponding to this dynamic turn in SLA research, more innovative qualitative research methods have emerged, which are capable of capturing the developmental nature of learner-or teacher-related affective variables longitudinally. Examples suggested by Hiver and Al-Hoorie (2019) for the SLA domain is qualitative comparative analysis, process tracing, concept mapping, agent-based modeling, retrodictive qualitative modeling, and social network analysis.

Attention to affective factors in SLA research goes back to the early 1970s, with the proposal of the Affective Filter Hypothesis by Dulay and Burt (as cited in Brown, 2001), which was later on completed by Krashen (1981). Teaching and learning the foreign/second language went beyond linguistic matters and the roles of psychology and, thus, affective factors were highlighted (Brown, 2001; Ni, 2012). For years, negative affective variables, referred to as affective filters (e.g., anxiety, demotivation, low self-confidence, and stress) were investigated in the SLA domain. However, with the advent of positive psychology in SLA research (MacIntyre and Mercer, 2014; MacIntyre et al., 2016), researchers have become more and more interested in exploring positive emotions (Wang et al., 2021, 2022), involved in L2 learning as well. Influenced by the CDST, longitudinal investigations of the developmental nature of teacher/learner-related affective variables in an L2 classroom are becoming more prevalent than before. More specifically, several qualitative studies of L2 affective variables have been conducted so far, but they are still limited in number and scope, as the SLA research has only recently entered its third phase of development (i.e., the dynamic phase) after the domain-general and domain-specific phases (Dewaele and Li, 2020). Exemplary works of qualitative research on L2 affective factors in the light of CDST are summarized in this review. The novelty of the emerging qualitative methods in recent explorations of L2 affective variables due to the dynamic turn in the of L2 affective domain might raise the question of the level of adherence of these methods to the basic evaluative criteria for qualitative research. That is, due to their incorporation of the properties of the CDST which are still new in terms of practice in the L2 affective domain, the qualitative CDST-compatible methods in this domain might face some misunderstandings in terms of their evaluation through the lens of the basic evaluative criteria.

It should be noted that this review paper does not imply that CDST-inspired research and the recently CDST-compatible methods used in the L2 affective domain should necessarily follow the basic principles of the basic evaluative criteria outside the field of SLA. Instead, it sparks a reflection on these criteria in these innovative methods to maximize their credibility. One might wonder why the innovative qualitative CDST-compatible methods used in the L2 affective domain are not quite consistent with the well-established evaluative criteria for qualitative studies outside the field of SLA. One reason might be due to the nature of CDST research with its own unique specificities. For instance, the self-organizing nature of complex systems might not provide learners with any clear research questions and justifications prior to data collection and data analysis. Some strong evidence regarding the justification of a CDST-qualitative research as well as the sufficiency of data might be achieved when the data is being analyzed. Also, the boundary of reflexivity or researcher bias due to this self-organizing property of CDST might not be always clear. Moreover, the process of data collection and data analysis might be intertwined. This point is justified by the feature of non-linearity in CDST (Hiver and Al-Hoorie, 2019) in the sense that in a CDST-compatible research procedure, the data collection and analysis does not have to be sequential or linear. Corresponding to the fluctuating nature of L2 affective variables, the data collection and analytic phases can be mixed and recursive, as has been often followed by L2 affective qualitative research in recent years.

It should be also mentioned that CDST adaptation in terms of methods has been noticeable in recent L2 affective research. Thus, the scope of this critical review is limited to the L2 affective domain and the points raised and discussed here cannot be expanded to the other domains of SLA. Given the context-bound, dynamic, and feed-back sensitive nature of dynamic systems, one might reckon whether some general criteria for the evaluation of the CDST-oriented qualitative studies on L2 affective factors can be expected or not. For instance, when it comes to the criteria of trustworthiness or credibility, some doubts might be casted on the application of these criteria in CDST-oriented methods. However, a closer look at these methods might provide us with common boundaries between the domain of these methods and the well-established evaluative criteria taken for granted for qualitative research.

## Exemplary dynamic qualitative research on L2 affective factors

The dynamic turn in the field of SLA, and the L2 affective domain in particular, under the influence of CDST and its related properties have encouraged researchers interested in this domain to apply innovative qualitative methods which are compatible with this meta-theory in their recent explorations of the L2 affective variables. Chan et al. (2014) employed retrodictive qualitative modeling, an innovative CDST-compatible research method to explore L2 learners’ motivation. They began their research project which was set in a Hong Kong junior high school, by initially asking a teacher focus group (another qualitative research method) to separate salient learner archetypes in the classes and, based on the teachers’ descriptive accounts, the researchers held in-depth interviews with the prototypical student in every group. They gained knowledge about the “signature dynamics” of the motivational system related to the individual prototypes. They showed the effectiveness of the retrodictive qualitative modeling in unraveling the causal mechanisms for a certain outcome related to language learners.

Hiver and Dörnyei (2017) investigated EFL teachers’ motivation and well-being. They used the retrodictive qualitative research method to explore how the EFL teachers kept their professional balance and professional efficiency in spite of their challenging and stressful workplace and in the face of all conflicting variables their job made them deal with. Hiver (2015) produced evidence for teachers’ development of a higher-order psychological entity called the “teacher immunity” as a reaction to conflicts specific to the classroom. To do this, he explored the dynamic patterns of these two outcomes (i.e., the adaptive teacher immunity outcome along with the maladaptive teacher immunity outcome) with the qualitative approach and managed to find the traces of the dynamic variation of the two hand in hand.

Elahi Shirvan and Talebzadeh (2020) admitted that the signature dynamics of language learners’ anxiety that emerged out of their language learning experience, as a negative emotion, and enjoyment, as a positive emotion, were not explored yet. Influenced by Dörnyei (2014), these researchers employed retrodictive qualitative modeling as an innovative approach to investigate the signature dynamics of these two learner-related factors. These researchers managed to identify the enjoyment and anxiety archetypes through focal-group interviews with a number of teachers about their students’ anxiety and enjoyment in practice. They held in-depth interviews with a prototypical language learner from every archetype so as to find about the trends and trajectories that led to a particular result or attractor state by tracing and exploring the dynamic events backward.

In the same year, Elahi Shirvan et al. (2020) conducted an ecological assessment of the dynamics of foreign language enjoyment in a time-based sampling scheme. They used open-ended interviews with English language learners at the intermediate level of proficiency for months. They also used journal writings for weeks. The results showed that enjoyment in foreign language changed temporally from moment to moment and also over months. Finally, these researchers discussed the emergent patterns of enjoyment among the different points of time according to the basic assumptions of the CDST.

In a more recent study, Yazdanmehr et al. (2021) used a process-tracing approach (another innovative far less applied CDST-compatible qualitative research method) to investigate the underlying causes of boredom in an online language learning course. Their case study was conducted on an adult learner of the German language whose accounts were qualitatively analyzed to trace variation in the boredom she experienced during the whole course. The longitudinal analysis of changes in her negative emotion during the entire course showed that the outset of the program was the most boring. Yet the language learner continued to feel bored till the end of the program but for different reasons. The process tracing approach proved to be an effective longitudinal qualitative research method in unwrapping the causal mechanisms accounting for the boredom grown in the online language course.

One of the most recent studies has been conducted by Shahnama et al. (2021) to investigate the stressful challenges an EFL teacher faced during an online English program of an intermediate level of proficiency. These researchers employed a process-tracing approach to discovering the causal mechanisms accounting for the troublesome distracting issues that occurred in the beginning, middle, and end of the language learning program. The results showed that inadequate technological facilities accounted for the most troublesome issues faced during the course, particularly in the beginning and middle of the program.

## The need for evaluative criteria for qualitative research

As mentioned previously, with the recent shift to the dynamic phase of research in SLA, more longitudinal qualitative studies are emerging in the field, especially to explore affective variables, which are of a developmental and dynamic nature. However, as the dynamic turn has only recently begun, the existing literature seems to be characterized by much heterogeneity and inconsistency in the use of qualitative research methods. For instance, several innovative qualitative methods such as process tracing, retrodictive qualitative modeling (Elahi Shirvan and Talebzadeh, 2020), the ididodynamic method (Boudreau), and the Q methodology have been recently used in the investigation of L2 affective variables but the application of the evaluation criteria in these studies have been neglected. Thus, there is a need for the consideration of specific evaluation criteria to increase the rigor and systematicity of the burgeoning body of qualitative research in L2 affective variables. Thus, the present study sought to derive these evaluative criteria from the domain-free sphere and summarize those that were more emphasized and best fitted the dynamic and domain-specific qualitative SLA research of affective variables.

It is not easy or all-agreed-on to decide what makes some qualitative research rigorous. However, evaluative criteria, firstly, depend on the qualitative research epistemological paradigm (positivist, interpretivist, and pragmatic), and, secondly, on the research method used for exploring the variables of interest. SLA research in recent years, influenced by the CDST, is more of the interpretive and pragmatic type see Dörnyei and Ryan (2015), and the new line of qualitative investigations, which aim to trace the developmental and temporal changes of the affective variables in recent years (as reviewed above) are mostly longitudinal, case studies or the like. As reviewed above, they included process writing, retrodictive qualitative modeling, ecological studies which entailed interviews, retrodictions, journal writings and even the use of innovative measurement instruments (e.g., enjoymeter and boredometer) which allowed for a continuous checking up on the participant’s momentary emotions. In this paper, we try to adapt the best fitting evaluation criteria for qualitative research to be compatible with the new dynamic shift in SLA research so as to be used by prospective researchers as standards of excellence to add rigor to their new inquiries of L2 affective variables.

Second language acquisition researchers should perceive the need to adhere to relevant evaluative criteria when conducting or evaluating some qualitative research. Relevant criteria should match or emerge from the same tradition, and research approach (data collection and analysis methods) which the researcher used or is planning to use. The evaluative criteria that we suggest for the dynamic longitudinal qualitative investigations of L2 affective factors have been derived from famous, highly cited published criteria by scholars in research methodology collected and categorized. These will be reviewed below.

## Review of evaluative criteria for qualitative research

Altheide and Johnson (1994) encouraged qualitative researchers to adopt an interpretive and pragmatic stance to research. They also favored the perspective of realism in analysis and the need to keep an eye on the goal of study in every step taken to conduct the research. Their approach was a social constructivist one, and they emphasized that a qualitative study should seek for meaning in communication, in dialogs, which can undoubtedly be much to the interest of SLA researchers, who show interest in exploring how L2 affective variables emerge out of classroom interactions and communicative tasks. Altheide and Johnson also highlight that the variable of interest should be investigated qualitatively in the very context it is embedded in, and not in isolation. This is also acknowledged in the CDST, with a focus on investigating different cognitive, affective, and behavioral patterns emerging out of the real network of variables interacting with each other in the immediate context (e.g., an L2 class). The other criterion set by Altheide and Johnson is the need to jot down as many details as possible about the context of research (e.g., the number of participants, physical features of class, the assigned roles to individuals, time scales, etc.). These scholars highly emphasize the authenticity of data, as they contend the experience should be recorded as it was actually lived by the participants. This authenticity seems to be to a large extent accommodated in the momentary records of emotions (e.g., enjoyment or boredom) *via* the innovative instruments (e.g., enjoymeter or boredometer) recently used in SLA research.

Britten et al. (1995) provided several criteria for evaluating qualitative research, which is summarized here as clear goal(s), clear implications, clear research questions, justification of the qualitative research method, (non)generalizability of findings, clear sampling strategy, clear methodology, statement of the potential effect of research method on data collection (reflexivity), systematic data analysis, the sufficiency of data for analysis, strongly evidenced conclusions, and reliable and valid data.

Some of these criteria seem to have been more adhered to in the existing qualitative studies of L2 affective variables in recent years, yet some not. Examples of the former are the clearly stated goals, implications, (non)generalizability of findings, and systematic data analysis. For others, there should be more cautious as, for instance, the methodology section especially the data analytic framework seems to lack clarity and reader-friendliness in some cases (e.g., Yazdanmehr et al., 2021) and requires simplification in writing so as to remove confusion for readers who might be totally stranger to the innovative approach (e.g., process tracing or retrodictive qualitative modeling) and may want to just get to know about the detailed procedures. In most of the existing literature, there is no mention of reflexivity, and no clear statement of the reliability and validity of the data used for analysis. More clarity is also needed for the elaborations on the systematic data analysis because the CDST compatible research methods are new and may need more details included in the research procedures.

Creswell (1998) suggested eight procedures to verify the findings of qualitative research and emphasized that any qualitative study use at least two of them. These include: prolonged exposure to the source of data and consistent observation, triangulated data, debriefing or peer review, negative case analysis, statement of researcher bias (reflexivity), member-checking, in-depth descriptions, and external audits. Also, Creswell emphasized that Lincoln and Guba’s three criteria of trustworthiness, credibility and authenticity should be adhered to in the evaluation of qualitative research. The existing dynamic qualitative studies of L2 affective variables seem to enjoy most of these evaluative criteria such as in-depth descriptions of data collection and analysis and triangulation. Yet, they seem to significantly lack some others such as reflexivity (except for the process tracing works of research), member-checking and external audits.

Elder and Miller (1995) criteria for rigorous qualitative research can be summarized as the need for: clear research questions, clear implications, clear research design, clearly-introduced participant(s), sampling type, detailed data collection and analysis, justified data collection and analysis methods, trustworthiness, and believability of reports. These are to a great extent similar to the evaluative criteria set by the previous scholars in research methodology, as reviewed. It seems that all the scholars so far have emphasized the clarity of all research procedures in qualitative studies. This makes even more sense to the CDST-led line of research on L2 affective factors as the recent studies are increasingly using complicated newly introduced or applied qualitative research methods. Thus, the novelty of the research procedure in the light of the CDST approach requires more comprehensive descriptions of procedural details, especially for reader researchers who may want to adopt a similar approach to replicate the research, or think of a similar research design.

To evaluate the rigor of qualitative research methods, Giacomini and Cook (2000) emphasized several aspects: fitness of research design to research questions, reasonable sampling, reasonable data collection and analysis procedures, sufficient data, in-depth data, clear statement of the co-occurring or the iterative type of data collection and analysis, sufficiently corroborated findings, recursive data analysis, and triangulation. What is new in Giacomini and Cook’s set of evaluative criteria for qualitative research, compared to the afore-mentioned criteria, is an emphasis on clarifying whether the data collection and analysis happened together or one followed the other, and also the need for recurrent return to the data to get further evidence (during the analysis). This point is justified by the feature of non-linearity in CDST (Hiver and Al-Hoorie, 2019) in the sense that in a CDST-compatible research procedure, the data collection and analysis does not have to be sequential or linear. Corresponding to the fluctuating nature of L2 affective variables, the data collection and analytic phases can be mixed and recursive, as has been often followed by L2 affective qualitative researchers in recent years.

Hammersley (1990) emphasized the validity and relevance of qualitative research. To ensure the former, this scholar suggested thinking twice about the match or mismatch between the available data, the evidence gathered and the claims made by the researcher. For the latter, he suggested making sure that the findings (even of case studies) can have public concerns, and be of value to the public. For example, the process tracing approach taken by Shahnama et al. (2021), despite being a case study, provided insightful predictions of sources of challenges in the online L2 courses held during the COVID-19 pandemic. The causal mechanism discovered could benefit all L2 teachers and learners experiencing language learning during the pandemic.

[Bibr B30][Bibr B30] evaluative criteria for qualitative research are considered as among the most commonly cited evaluative framework, with the trustworthiness of research at its core. Lincoln and Guba introduced four aspects of trustworthiness: credibility, transferability, dependability, and confirmability. Credibility means being sure of the truth of findings. Transferability means being sure that the findings can be used in other similar contexts. Dependability means being sure of the consistency of the findings if replicated in the same setting. Confirmability means being sure that the least bias was involved in the findings. To increase credibility, for example, qualitative researchers are suggested to do triangulation or peer-check. The former seems to be more prevalent in qualitative studies of L2 affective variables in the dynamic era of SLA research. For instance, Elahi Shirvan et al. (2020) used interviews, journal writing and emotion-meter all together for collecting and analyzing data. The technique Lincoln and Guba’s (1985) suggested for increasing transferability is the use of thick descriptions, which is also common in all the existing qualitative body of dynamic L2 affective research in recent years. To increase dependability, researchers are suggested to use external audit (asking another researcher to come and analyze the findings and judge the quality of interpretations), which seems to be significantly absent in the existing body of qualitative research of L2 affective variables in recent years. Reflexivity and triangulation and several other techniques are suggested to increase confirmability, among which triangulation seems to be more common in SLA research.

Malterud (2001) evaluative criteria for qualitative research are well-adapted to the CDST, as they emphasize the consideration of context and going beyond universal claims. What Malterud suggests to take into account in evaluating qualitative research include reflexivity, transferability, giving detailed descriptions of the research context, going beyond the mere data to make interpretations and to consider the relations between the data and the theoretical knowledge, and providing clear details on data coding and pattern extraction. Among these criteria, some have been mentioned before in the evaluative criteria set by other scholars such as reflexivity, transferability, and the need for detailed descriptions of research procedure. What is new seems to be the need for linking the data-based findings to the related theories, which has been done to different degrees in the existing dynamic qualitative L2 affective factors literature, depending on the methodology used. For example, the process tracing studies (e.g., Shahnama et al., 2021; Yazdanmehr et al., 2021) have done so because an obligatory step defined in the process tracing approach is to find theoretical justifications for the state of a certain affective variable at every temporal phase of study.

(Mays and Pope, 1995, 2000a,b) contended that in qualitative research rigor is guaranteed by having a systematic and self-aware research design, data elicitation, analysis, and generalization of findings (if any). This can improve the reliability of the results. Among the evaluative criteria they set are: the need for adequately introducing the variable under study, clearly describing the data collection and analysis procedure, contribution of the research findings to the existing knowledge, and the extent to which the researcher considered the effect of the research method used on the findings obtained (reflexivity). Attention to detail in the context of research and the network of interactions affecting and being affected by the variable of interest have already been significantly considered in the CDST. Thus, this set of criteria also seems to be justified for the dynamic turn of SLA research. As the research methods used have been mostly innovative, the authors of the existing literature on the dynamic qualitative research of L2 affective variables have included detailed step by step accounts of the data collection and analysis procedures.

The evaluative criteria set by Miles and Huberman (1994) are five in number. These include objectivity (least biased), reliability or dependability, validity and authenticity, transferability, and application (implications). The viewpoint seems to be to a great extent pragmatic, as Miles and Huberman (1994) greatly emphasizes the practical implications of the research findings and the usefulness of research, which needs to be clearly stated in the research. In the existing literature on the dynamic qualitative research of L2 affective variables, the implications are included in some studies both in the abstract and the ending section of the papers, and in some others only in the ending part of the manuscripts and not in the abstracts. Probably, it can be suggested to include the implications as an obligatory move in the abstract too so as to ensure readers that what has been done has something to offer to the SLA domain and is to benefit L2 learning in a way or more.

Patton (1999, 2001) draws attention to the credibility of the qualitative research. He suggests that rigorous data collection and analysis techniques be used to ensure high-quality data. He pinpointed the reliability, validity and authenticity of data, and the essentiality of the researcher’s being experienced in doing this type of inquiry or having had the relevant training to do so. Patton also emphasizes the need for creative and innovative data analytic procedures, and also the need for providing comprehensive details about the research procedure. What seems to be new in Patton’s criteria is the emphasis on well-trained researchers. This is quite defendable in the dynamic phase of SLA research, marked by an array of innovative research methods that are compatible with the CDST. The researchers are expected to read profusely about the procedures of each new qualitative research method (e.g., process tracing, retrodictive qualitative modeling, ecology of education) and even mixed designs (Hiver and Al-Hoorie, 2019).

In another set of evaluative criteria for qualitative research, Popay et al. (1998) suggest several dimensions: use of adequately representative data, attention to the social context of research and flexibility of research design, reasonable sampling, adequate descriptions, evidence for data quality, adequate links to theories, evaluating the typicality of the findings. There seems to be an emphasis, in this set of evaluative criteria, on the social context of data collection and analysis. Attention to the socially constructed and even transmitted affective variables within the classroom learning see Frenzel et al. (2018) is admitted in CDST too. Thus, SLA researchers are expected to consider the effect of these social interactions in their analysis and interpretation of data. As for the flexibility suggested by Popay et al. (1998), one suggested technique is triangulation of data gathering, which as mentioned previously, has been to a great extent obeyed by qualitative researchers of L2 affective variables in recent years.

Finally, Yardley (2000) draws qualitative researchers’ attention to sensitivity to context and comprehensive data collection, analysis, and interpretation. The former seems to be well fit in with the CDST turn in SLA research. The latter is the same idea put forth by all the aforementioned scholars suggesting some evaluative criteria for qualitative research. Most of the existing qualitative literature on L2 affective variables is case studies, for which the context-sensitivity issue is ever more important. So is the need for comprehensive descriptive accounts to facilitate meaning-making and interpretations. The other two important criteria set by Yardley (2000) are reflexivity and practical implications of findings, as already discussed in the preceding sets of criteria.

## Common evaluative criteria for dynamic qualitative research on L2 affective variables

The most commonly used evaluative criteria for qualitative research suggested by the mostly cited scholars in research methodology were reviewed in the previous section and their relevance to the dynamic qualitative studies of L2 affective variables was discussed. A glance at the basic evaluative criteria for qualitative research reviewed above indicates that most of the suggested criteria overlap in nature but do not have the same labels. There were many commonalities among these criteria, which can be summarized here to guide prospective SLA researchers interested in the CDST compatible line of inquiry into L2 affective variables. These include the authenticity of research, justified research method, reasonable sampling, clear and detailed descriptive of the social context of research (the setting), detailed data collection and analysis procedures, links to theoretical backgrounds, reflexivity, triangulation, reliability and validity, and clear implications.

As already discussed in the previous section, some of these criteria seem to have been adhered to, more than others, so far in the related literature of L2 affective variables. For example, detailed data collection and analysis has been adhered to in all the existing qualitative dynamic studies of L2 affective variables. More specifically, some evaluative criteria like Yardley (2000) with a focus on context sensitivity in the qualitative research can be more reflective of the dynamic nature of the recent emerging qualitative methods in the L2 affective domain. That may be because the CDST compatible research methods are mostly innovative, and the procedures should not be taken as evident for readers. The readers should not be expected to have to refer to other sources to get to know the basics of the new research method (e.g., process tracing). The author needs to state in clear and reader-friendly accounts how s/he precisely collected and analyzed the data step by step. Triangulation is another suggested technique to add rigor to the qualitative research, as agreed by almost all the scholars whose suggestions were reviewed above. This seems to be also to a large extent present in the existing qualitative dynamic studies of L2 affective variables. Triangulation improves research reliability and validity.

One criterion that has been less adhered to in the existing qualitative dynamic studies of L2 affective variables seems to be the justification for the use of a particular research method. There seems to be a need for bringing more evidence on the distinctive benefits of the selected research method and persuade the readers why a certain method (e.g., retrodictive qualitative modeling) was preferably used among other qualitative methods. Another criterion which has been occasionally absent in the existing qualitative dynamic studies of L2 affective variables is reflexivity. The authors are expected to clearly state how they made sure they minimized the biased responses. This reflexivity can be currently only guaranteed in the process tracing approach to qualitative research, as it is one of the obligatory steps specified within this approach, which makes the author carefully deal with it and also quote it clearly in the study. Yet, in other research methods, the researcher him/herself is supposed to state the bias-shooting measures clearly in the methodology section of the manuscript. There seems to be also a need for further attention to the theoretical roots of the research findings. Some qualitative research methods such as the process tracing have this step involved in its overall analytic framework. In other words, the researchers are supposed to find both theoretical and non-theoretical explanations for the fluctuations in the affective variables under study. Yet, not all the existing qualitative dynamic studies of L2 affective variables adopted a process-tracing approach, and not all committed themselves to bring theoretical reasons for the emergent patterns of change in the affective variable of interest.

Finally, as already discussed in the previous section, there is a strong emphasis on clearly stating the practical implications of the study in qualitative research. These are expected to be stated reasonably, authentically and in detail in the research manuscripts and even in the research proposals. As the current line of qualitative research in the world tends to follow a pragmatic paradigm, stating the contributions of the research findings to the real world is of an utmost importance. In SLA research, this should be subsumed under the pedagogical implications of the study. Though all the existing qualitative dynamic studies of L2 affective variables include pedagogical implications, authors are recommended to have them mentioned in the abstract too. Yet, currently, implications are conspicuously absent or just superficially tapped onto in the abstract. The readers are delayed to see the pedagogical implications of study in the ending part of the published works of research.

## Evaluative criteria arguments with an example of an empirical study in the L2 affective domain using an emerging qualitative method

Due to their innovative nature, the recent studies using emerging qualitative methods in the L2 affective domain have mainly expanded their explanation regarding the method. However, as these explanations are aimed to clarify the reasons for the use of the new method and a systemic presentation of its procedures, they might seem somehow inconsistent with the expectations of basic evaluative criteria. This point can be drawn by a reflection on Kruk et al.’s (2022) Q-methodology for the exploration of potential sources of boredom in learning a foreign language. Given the innovative nature of Q methodology in the L2 affective domain, the researchers have elaborated on an integration of data collection and data analysis in terms five steps. Such explanations might render them away from the simplifications expected by some of the basic evaluative criteria. Thus, the explanations provided by Kruk et al. (2022) in the data for the steps of data collection and data analysis might not be simple to understand at the first glance but these explanations are necessary for the sake of readers’ full comprehension of how the method functions in case they should need to replicate the same procedures in another study. Moreover, the integration of data collection and data analysis is in alignment with the non-linearity principle of CDST framework see Hiver and Al-Hoorie (2019) as a linear path from data collection to data analysis might not be necessarily expected.

Although there is no explicit section in this study about trustworthiness, transparency, and credibility but they have been addressed in the five step-procedures provided for data collection and data analysis. That is, the main criteria of justification of research method, clear description of the setting of the study, detailed explanation about data collection and data analysis as well as connections with the theoretical backgrounds of the study have been covered. Like most of qualitative studies in the L2 affective domain, there is also no mentioning of reflexivity and external audits in this study but member checking as a reflection to the current data for the achievement of strong evidence has been conducted. On the other hand, the procedures of the use of Q methodology by Kruk et al. (2022) are quite in line with Malterud’s emphasis on the contextual consideration of a given study. To do this, the association of the collected findings with the main underpinning theories of boredom has been meticulously carried out. It is worth noting that this consideration of the contextual specificities is not an obligatory phase of the Q methodology but it has been incorporated during the data analysis phase by the researchers of the study themselves. This endeavor also reflects Yardley’s criterion of context sensitivity, which is quite compatible with the principles of CDST. Besides, the authors of the study seem literate enough for conducting the study. This is what Patton emphasized as an important criterion for conducting qualitative research.

## Conclusion

Qualitative research of affective factors is subsumed under the psychology of education, with the aim of investigating how psychosocial processes are formed by all the people, their interactions, emotions and behaviors that comprise their ever-fluctuating context (making up the research context) (Yardley, 2017). A main advantage of qualitative research is that it can explore and measure contextual factors; thus, it can be marked by context-sensitivity by taking into account the participants’ points of view and setting, the sociocultural and linguistic context of the study, and how they can affect how participants feel and how this feeling is captured and interpreted by the researcher (Yardley, 2017). This is the value of exploring affective variables through qualitative research. Yet, the present study pinned down the SLA domain and the new line of research enlightened by the CDST. It focused on the qualitative studies of L2 affective variables in recent years led by the dynamic approach. Indeed, the new line of research has employed innovate research methods compatible with the CDST and has had useful findings. Yet, they seem to lack in rigor and systematicity of research. This observed lack of consistency was problematized in this study, and attempts were made to set evaluative criteria for the judgment of the burgeoning studies and guiding the future line of inquiry. To this aim, the different sets of evaluative criteria proposed for qualitative research in general were derived from the mostly cited scholars in research methodology. The relevance of each to the dynamic qualitative investigations of L2 affective variables was discussed. Then these evaluative criteria were summarized and put forth for the qualitative research in the dynamic phase of SLA research. The overall goal was to guide future researchers with an interest in investigating the dynamic developmental nature of L2 affective variables qualitatively. This review study implied that despite the innovation of CDST-oriented qualitive research in the L2 affective domain, it does not seem that these studies have violated their adherence to the basic criteria for the evaluation of qualitative research. Also, some criteria are more suitable than the other ones for the incorporation the CDST approach. These evaluative criteria can pave the way for the emergence of more rigorous and systematic qualitative studies of L2 affective factors in upcoming years. As reviewed in this study, despite some differences due to the nature of complex systems such as non-linearity, feedback-sensitivity, and change, the procedures of evaluation of the CDST-oriented research might not be a fully consistent with those of the basic evaluative criteria of qualitative research. But this does not mean that there is hardly any match between qualitative CDST-compatible methods and those of the basic evaluative criteria as most of these studies have represented the main evaluative criteria. Thus, description of a prototypical paper reflecting the basic evaluative criteria was not within the scope of this review. Instead, how the adherence to these criteria can be made *via* the use of these methods was the aim of the study.

Some points of criticism have been sometimes made against CDST qualitative research for their lack of adherence to the basic evaluative criteria of qualitative studies. As this review indicates, this is not always the case in the L2 affective domain. Mos of the CDST-adapted methods in this domain have overlaps with these criteria but the discrepancies are rooted in the unique purpose of each study. Thus, researchers with a CDST-interest in the L2 affective domain are suggested that they should be aware of these evaluative criteria in their qualitative research and make the required adaptations in these criteria based on the unique features of their studies. A challenge researchers might face in the practice of CDST-led qualitative research on L2 affective variables is how to adapt the basic evaluative criteria into their CDST orientation. However, the review of these criteria in this study indicate that this endeavor should not be seen demanding. A careful consideration of the purpose of a given study, accounting for a specific qualitative method from a CDST perspective (e.g., process tracing and qualitative comparative analysis) and the procedures of the selected method to follow that purpose can pave the way for the incorporation of the evaluative criteria. In other words, having the basic criteria for the evaluation of qualitative research in mind, the researchers interested in the L2 affective domain are supposed to check the adherence of the innovative methods they use in their future studies to these basic criteria. More specifically, paying meticulous attention to the basic evaluative criteria for qualitative research and a reflection on these criteria in the use of the emerging innovative methods in the L2 affective domain can strengthen the adherence of these methods in the future studies to these criteria.

## Author contributions

The author confirms being the sole contributor of this work and has approved it for publication.
